# Functional Characterizations of Chemosensory Proteins of the Alfalfa Plant Bug *Adelphocoris lineolatus* Indicate Their Involvement in Host Recognition

**DOI:** 10.1371/journal.pone.0042871

**Published:** 2012-08-10

**Authors:** Shao-Hua Gu, Song-Ying Wang, Xue-Ying Zhang, Ping Ji, Jing-Tao Liu, Gui-Rong Wang, Kong-Ming Wu, Yu-Yuan Guo, Jing-Jiang Zhou, Yong-Jun Zhang

**Affiliations:** 1 State Key Laboratory for Biology of Plant Diseases and Insect Pests, Institute of Plant Protection, Chinese Academy of Agricultural Sciences, Beijing, China; 2 Department of Biological Chemistry, Rothamsted Research, Harpenden, United Kingdom; Ghent University, Belgium

## Abstract

Insect chemosensory proteins (CSPs) have been proposed to capture and transport hydrophobic chemicals from air to olfactory receptors in the lymph of antennal chemosensilla. They may represent a new class of soluble carrier protein involved in insect chemoreception. However, their specific functional roles in insect chemoreception have not been fully elucidated. In this study, we report for the first time three novel CSP genes (*AlinCSP1-3*) of the alfalfa plant bug *Adelphocoris lineolatus* (Goeze) by screening the antennal cDNA library. The qRT-PCR examinations of the transcript levels revealed that all three genes (*AlinCSP1-3*) are mainly expressed in the antennae. Interestingly, these CSP genes *AlinCSP1-3* are also highly expressed in the 5^th^ instar nymphs, suggesting a proposed function of these CSP proteins (AlinCSP1-3) in the olfactory reception and in maintaining particular life activities into the adult stage. Using bacterial expression system, the three CSP proteins were expressed and purified. For the first time we characterized the types of sensilla in the antennae of the plant bug using scanning electron microscopy (SEM). Immunocytochemistry analysis indicated that the CSP proteins were expressed in the pheromone-sensitive sensilla trichodea and general odorant-sensitive sensilla basiconica, providing further evidence of their involvement in chemoreception. The antennal activity of 55 host-related semiochemicals and sex pheromone compounds in the host location and mate selection behavior of *A. lineolatus* was investigated using electroantennogram (EAG), and the binding affinities of these chemicals to the three CSPs (AlinCSP1-3) were measured using fluorescent binding assays. The results showed several host-related semiochemicals, (*Z*)-3-hexen-1-ol, (*E*)-2-hexen-1-al and valeraldehyde, have a high binding affinity with AlinCSP1-3 and can elicit significant high EAG responses of *A. lineolatus* antennae. Our studies indicate the three antennae-biased CSPs may mediate host recognition in the alfalfa plant bug *A. lineolatus*.

## Introduction

The number of insect species on the earth, even at a conservative estimate, exceeds one million, which are far more than any other kind of living creatures [1], [2]. The prosperity of insect empire benefits from their effective chemical communication between individuals and with their environment, which is primary essential for mating and reproduction. The alfalfa plant bug *Adelphocoris lineolatus* (Goeze) (Hemiptera: Miridae) is a well-known pest in Europe, United States and China. This plant bug and several other mirids are extremely herbivores and cause severe damage to many important crops such as beans, strawberries, peaches, cotton, and various seed crops each year [3]–[5]. These mirids are attracted to flowering plants especially cotton, alfalfa and mung bean [3], [4], [6]–[9], suggesting mirids use chemical information from these flowers to forage suitable hosts and find oviposition sites. Identification of attractant molecules and their interactions with olfactory proteins are meaningful for monitoring and mass-trapping these mirids and other insect pest [10], [11].

Insects use olfaction, vision and audition to perceive environmental signals such as sound, fluorescence, supersonics and semiochemicals (plant volatiles and insect pheromones) [12]–[14]. However, the vision and audition systems in many insect species are poorly developed, so the olfaction system in insect is primary sensitive and sophisticated. There are two small soluble olfactory protein families, odorant binding proteins (OBPs) and chemosensory proteins (CSPs) in the chemosensory lymph between antennal cuticle and olfactory receptors. They are proposed to play an important role in the insect chemoreception by capturing and transporting hydrophobic chemicals from the environment to the chemosensory receptors [15]–[19]. However, their specific functional roles in insect chemoreception have not been fully elucidated. The evidences on their specificity and molecular recognition of semiochemicals are still lacking. Especially for insect CSPs there are some debates whether they are involved in insect olfaction and chemical perception.

Insect CSPs are also known as OS-D-like proteins [20] or sensory appendage proteins (SAPs) [21] before being named as CSPs because of their high expression in the antennae of the desert locust *Schistocerca gregaria* [16]. They are broadly expressed in various chemosensory organs, such as antennae [16], [19], [22]–[24], maxillary palps [25], labial palps [25], [26] and proboscis [27]. However, they are also found in non-chemosensory organs, such as legs [28], [29], wings [30]–[31] and pheromone glands [22]. There are no CSPs found in any of other animals [32]. CSPs may represent a new class of soluble carrier proteins involved in insect chemoreception. There are some main differences between OBPs and CSPs. (1) CSPs (10–15 kDa) are smaller than OBPs (15–20 kDa). (2) There are four highly conserved and structurally important cysteines in the CSPs, while the number of such cysteine residues in OBPs is six. (3) OBPs are mainly found antennae specific, while CSPs are found in the antennae, maxillary palps, labial palps, proboscis as well as wings and legs. (4) CSPs in diverse insect species show high amino acid identity, while OBPs have much lower amino acid identity (with an average of only 14%). (5) The 3D structures of CSPs and OBPs are both consisted of six a-helices connected by α-α loops. The four conserved cysteines in CSPs are connected by two pairs of non-interlocked disulphide bridges [18], while the six conserved cysteines in OBPs are paired in three interlocked disulphide bridges [33], [34].

To elucidate the molecular recognition of CSPs and examine their involvement in olfactory coding, in this study, three new CSP genes (*AlinCSP1-3*) in The alfalfa plant bug *A. lineolatus* were identified, the tissue and developmental distributions of the transcripts of *AlinCSP1-3* were measured by qRT-PCR, the different types of sensilla were characterized and the specific sensillum location of the CSPs AlinCSP1-3 in different sensilla was investigated by immunocytochemistry methods. The antennal activity of 55 host-related semiochemicals and sex pheromone compounds in the host location and mate selection behavior of *A. lineolatus* was investigated using electroantennogram (EAG), and their binding affinity to AlinCSP1-3 proteins were measured using fluorescent binding assays. Using homology modeling methods, the 3D structure of AlinCSP1-3 protein was constructed and the potential binding sites are discussed. Our studies provide further detailed evidences for the involvement of CSPs in insect chemoperception and host locations.

## Results

### Expression Profile of AlinCSP1-3 Transcripts

**Figure 1:**
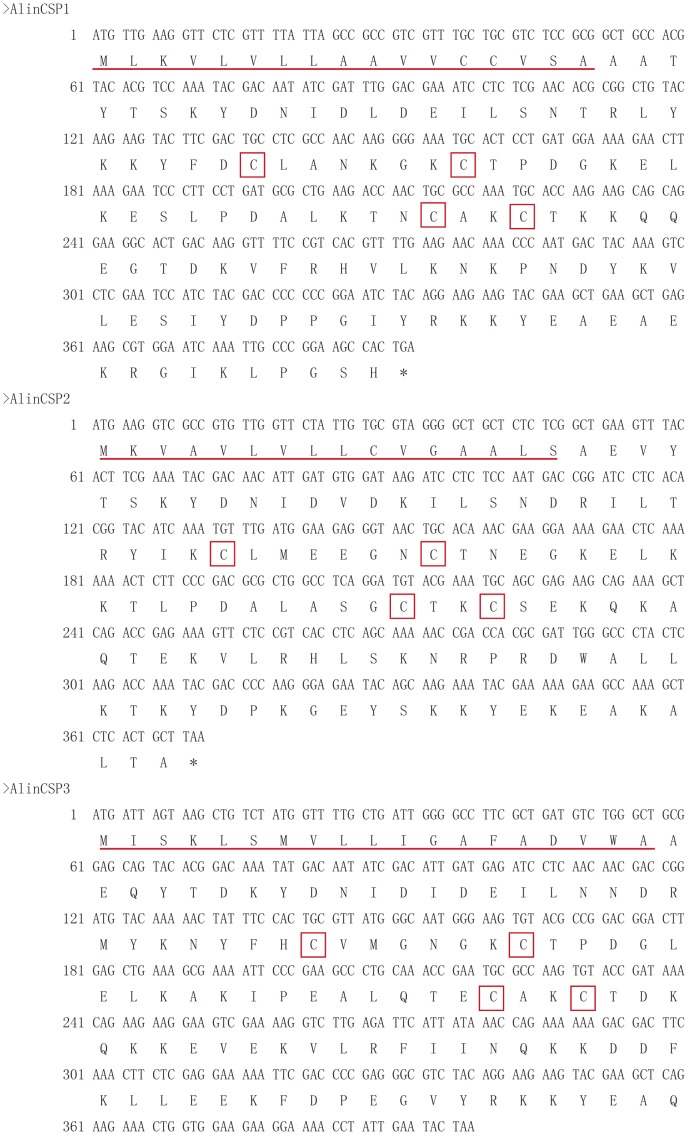
cDNA and predicted amino acid sequences of the CSP genes *AlinCSP1, AlinCSP2* and *AlinCSP3* of the alfalfa plant bug *A. lineolatus*. The N-terminal signal peptide sequences are underlined. The stop codon is indicated with an asterisk. Four conserved cysteines are showed with red boxes.

**Figure 2:**
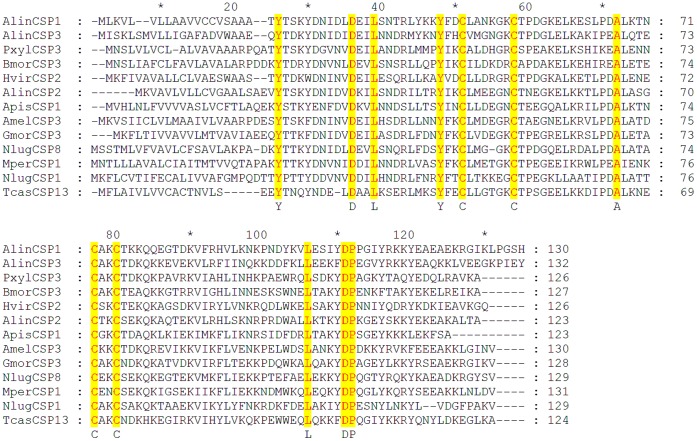
Alignment of peptide sequences of the alfalfa plant bug *A. lineolatus* CSPs, AlinCSP1, AlinCSP2 and AlinCSP3 with those of other insect species. Full-length amino acid sequences are aligned by ClustalX 1.83. Yellow colors show the four conserved cysteine and other conserved residues in the alignment. The other insect species are: *Plutella xylostella* (Pxyl), *Bombyx mori* (Bmor), *Heliothis virescens* (Hvir), *Acyrthosiphon pisum* (Apis); *Apis mellifera* (Amel); *Glossina morsitans morsitans* (Gmor); *Nilaparvata lugens* (Nlug); *Myzus persicae* (Mper); *Tribolium castaneum* (Tcas). GenBank accession number for all CSP genes are: AlinCSP1-3 GQ477014-GQ477016; PxylCSP3, EF202828; BmorCSP3, DQ855509; HvirCSP2, AY101511; ApisCSP1, NM_001134932; AmelCSP3, NM_001011583; GmorCSP3, FN432803; NlugCSP1, HM489006; NlugCSP8, FJ387497; MperCSP1, FJ387490; TcasCSP13, NM_001045816.

We have identified three new CSPs from the plant bug *Adelphocoris lineolatus* by constructing and screening the antenna specific cDNA library and named as *AlinCSP1*, *AlinCSP2* and *AlinCSP-3* (GenBank No. GQ477014-GQ477016). The *AlinCSP* genes contain an open reading frame (ORF) of 393 bp, 372 bp and 399 bp, respectively. The predicted amino acid sequences of *AlinCSP1-3* CDS have the typical four-cysteine signature of insect CSPs [18] with a signal peptide of 17, 16 and 19 amino acid residues at the N terminus, respectively (Figure 1), all the CSPs showed a common cysteine sequence motif of C_1_-X_6-8_-C_2_-X_16-21_-C_3_-X_2_-C_4_ [32]. The calculated molecular masses of mature AlinCSP1-3 proteins were 12.91 kDa, 12.31kDa and 13.38 kDa, respectively. The calculated isoelectric points of mature AlinCSP1-3 were 9.07, 9.11 and 5.57, respectively. The amino acid identity among the AlinCSP1-3 and other insect species CSPs is about 55% (Figure 2).

**Figure 3:**
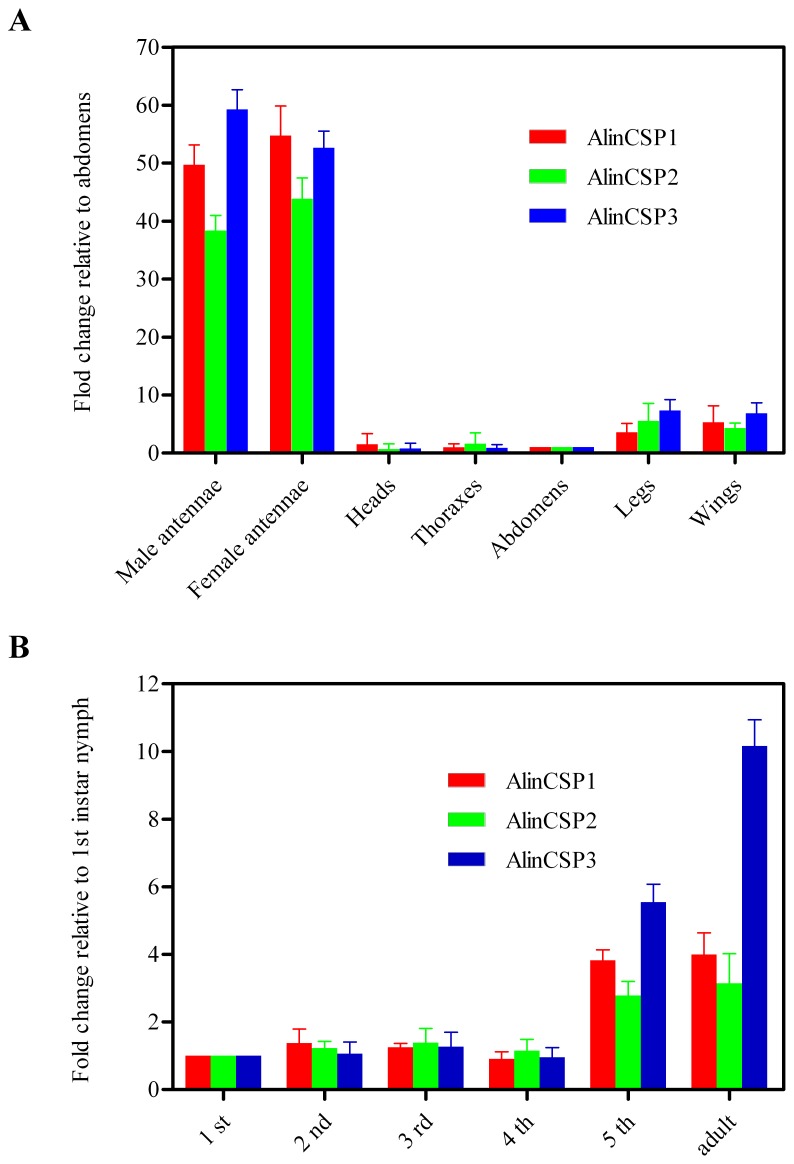
The transcript levels of the alfalfa plant bug *A. lineolatus* CSP genes evaluated by qRT-PCR. (A) different adult tissues and (B) different development stages The standard error is represented by the error bar.

The expression level of *AlinCSP1-3* transcripts in each adult tissues and development stages were measured by qRT-PCR with two internal controls, β-actin and elongation factor. The qRT-PCR results with the β-actin gene are shown in Figure 3 and similar results were obtained with the elongation factor (data not show). In general, the *AlinCSP1-3* transcripts were mainly expressed in the antennae. *AlinCSP1-3* showed very low expression level in the head, thorax and abdomen, legs and wings (Figure 3A). In different development stages, the *AlinCSP1-3* was mainly expressed in the 5^th^ instar nymph and adult stages, very low expression of three CSP genes was detected in the 1^st^–4^th^ nymph stages (Figure 3B).

### Antennal Sensilla of *A. lineolatus*

**Figure 4:**
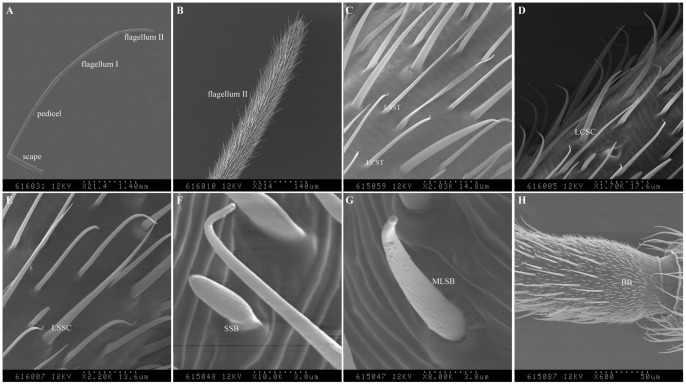
Scanning electron micrographs of the sensilla of *A. lineolatus* male adult antennae. (A) *A. lineolatus* antennae are about 6 mm in length and consist of scape, pedicel, flagellum I and II. (B) The flagellum II of *A. lineolatus* antennae. (C) Long curved sensilla trichodea (LCST) and long straight sensilla trichodea (LSST) on flagellum II. (D) Long curved sensilla chaetica (LCSC) on flagellum I. (E) Long straight sensilla chaetica (LSSC) on pedicel. (F) Short sensilla basiconica (SSB) and (G) Medium long sensilla basiconica (MLSB) on flagellum II. (H) Böhm bristles (BB) on the base of pedicel. Similar results observed in the female antennae. The number of each type hair is no statistical difference between sexes. The scale bars are indicated at the bottom of each figure.

The antennae of *A. lineolatus* are about 6 mm in total length and consist of three segments, including scape, pedicel, flagellum I and II (Figure 4A–B). There are four different kinds of sensilla on the male and female antennae, sensilla trichodea (ST), sensilla chaetica (SC), sensilla basiconica (SB) and Böhm bristles (BB). No statistical difference in sensilla numbers between sexes (Figure 4C–H).

The ST sensilla were the main type of both male and female antennae and can further subdivided into long curved sensilla trichodea (LCST) and long straight sensilla trichodea (LSST). LCST sensilla were mainly present in flagellum II (Figure 4C), this type of sensilla were long, curved inward with the tip, smooth and with no raised socket at the base. LSST sensilla were the most numerous type sensilla in both sexes and mainly present on the pedicel, flagellum I and II. This type of sensilla was long, slender, smooth and with no raised socket at the base (Figure 4C). Sensilla trichodea (ST) had thick cuticular walls with multiple pores on the cuticular surface and 2–3 nerve cells, which observed in the following immunolocalization experiments using transmission electro micrograph (TEM), similar feature was also reported in the tarnished plant bug, *Lygus lineolaris* [35].

Sensilla chaetica (SC) also had two types, long curved sensilla chaetica (LCSC) and long straight sensilla chaetica (LSSC). LCSC sensilla were mainly distributed on the flagellum I, this type of sensilla curves at the tip and with a raised socket at the base (Figure 4D). LSSC sensilla were mainly present on the scape and pedicel, few numbers were also found on the flagellum (Figure 4E). Sensilla chaetica (SC) was straight, grooved and a raised socket at the base. Both types had thick cuticular well, which observed in the immunolocalization experiments (see below).

Two kinds of sensilla basiconica (SB), short sensilla basiconica (SSB) and medium long sensilla basiconica (MLSB), were distributed on pedicel, flagellum. These hairs had blunt tips, grooves on the hair surfaces, no socket at the base (Figure 4F–G). Cross section of sensilla basiconica (SB) showed a thin cuticular well and 3–5 nerve cells. Similar types were also observed in the tarnished plant bug, *Lygus lineolaris* [35]. Böhm bristles (BB) was similar with LSSC in shape but much shorter in length, they were often present as clusters in the scape and pedicel (Figure 4H). The fine structure of Böhm bristles (BB) has been poorly documented previously.

### Immunocytochemistry of AlinCSP1-3

**Figure 5:**
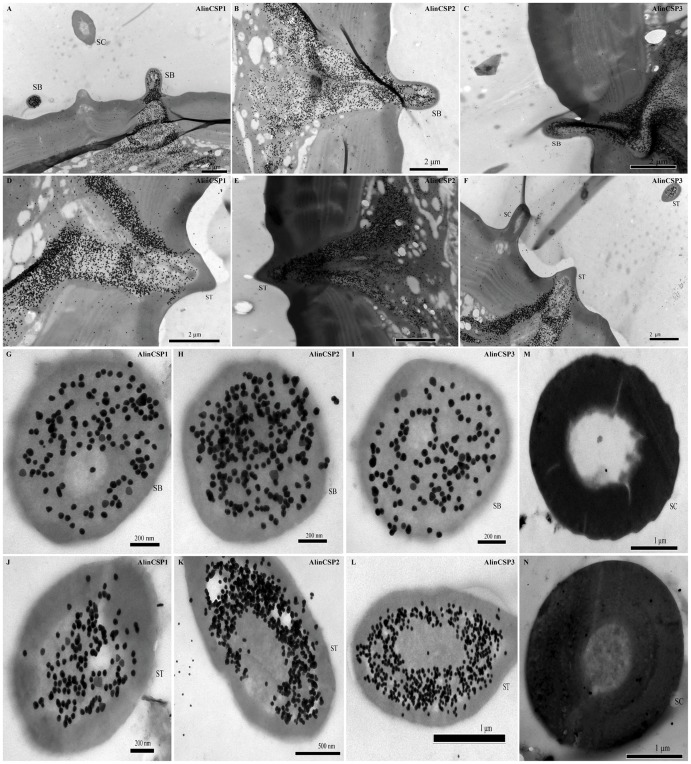
Immunocytochemical localization of the CSP proteins in the sensilla of the male adults of the alfalfa plant bug *A. lineolatus.* All three CSPs AlinCSP1, AlinCSP2 and AlinCSP3 were heavily labeled in the sensilla basiconica (SB) (the longitudinal sections A-C and the cross sections G-I) as well as in the pheromone-sensitive sensilla trichodea (ST) (the longitudinal sections D-F and the oblique section J-L). But the sensilla chaetica (SC) was not labeled (A, F, M and N). The few grains found in over the cuticle and the dendrites represent non-specific background. Dilution of primary antibody was 1∶5000 for AlinCSP1, 1∶10000 for AlinCSP2 and AlinCSP3. Secondary antibody was anti-rabbit IgG conjugated with 10 nm colloidal gold granules at a dilution of 1∶20.

Polyclonal antiserums were used for the cellular localization of the AlinCSP proteins in antennal sensilla of *A. lineolatus*. In the sections of different chemosensory sensilla, gold particles labeled the pheromone-sensitive sensilla trichodea (ST) and general odorant-sensitive sensilla basiconica (SB) (Figure 5), suggesting the protein expression of the CSPs in these sensilla. However, the sensilla chaetica (SC) was never labeled. The sensillum lymph in the sensillum hair lumen and the cavity below the hair base were heavily labeled (Figure 5A–L). While the dendritic cytoplasm and cuticles of the hair wall were never labeled (Figure 5M–N). The cellular localization of AlinCSP1-3 was similar between male and female antennae.

### Fluorescence Binding Assays

**Figure 6:**
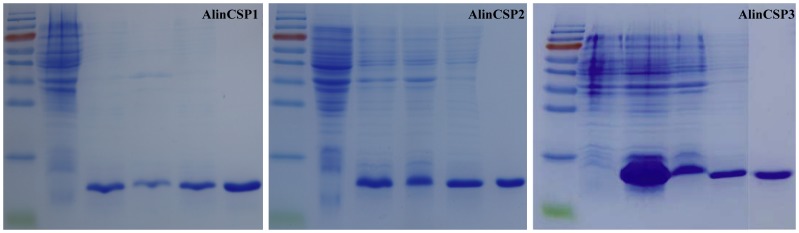
Expression and purification of AlinCSP1-3 proteins. The SDS-PAGE analyses of the recombinant CSP proteins; AlinCSP1, AlinCSP2 and AlinCSP3. Lane 1: non-induced protein, Lane 2: induced protein, Lane 3: supernatant, Lane 4: inclusion bodies and Lane 5: purified protein. Protein molecular weight marker (M), from the top: 170, 130, 95, 72, 55, 43, 34, 26, 17, 10 kDa.

To determine the binding affinities of the AlinCSP proteins to semiochemicals, we expressed *AlinCSP1-3* genes in a bacterial system and purified the recombinant proteins by a combination of anion-exchange chromatography and gel filtration. The size and purity of the recombinant proteins was examined by SDS-PAGE (Figure 6) and mass spectrometric analysis (Figure S1).

The fluorescence displacement assays were performed using a fluorescence probe N-phenyl-1-naphthylamine (1-NPN). When 1-NPN probe was bound to AlinCSP1-3 proteins (5 µM) and excited at 337 nm, its fluorescence emission peak shifted from 460 nm to 430 nm, accompanied by a several time increase in intensity (Figure S2).

**Figure 7:**
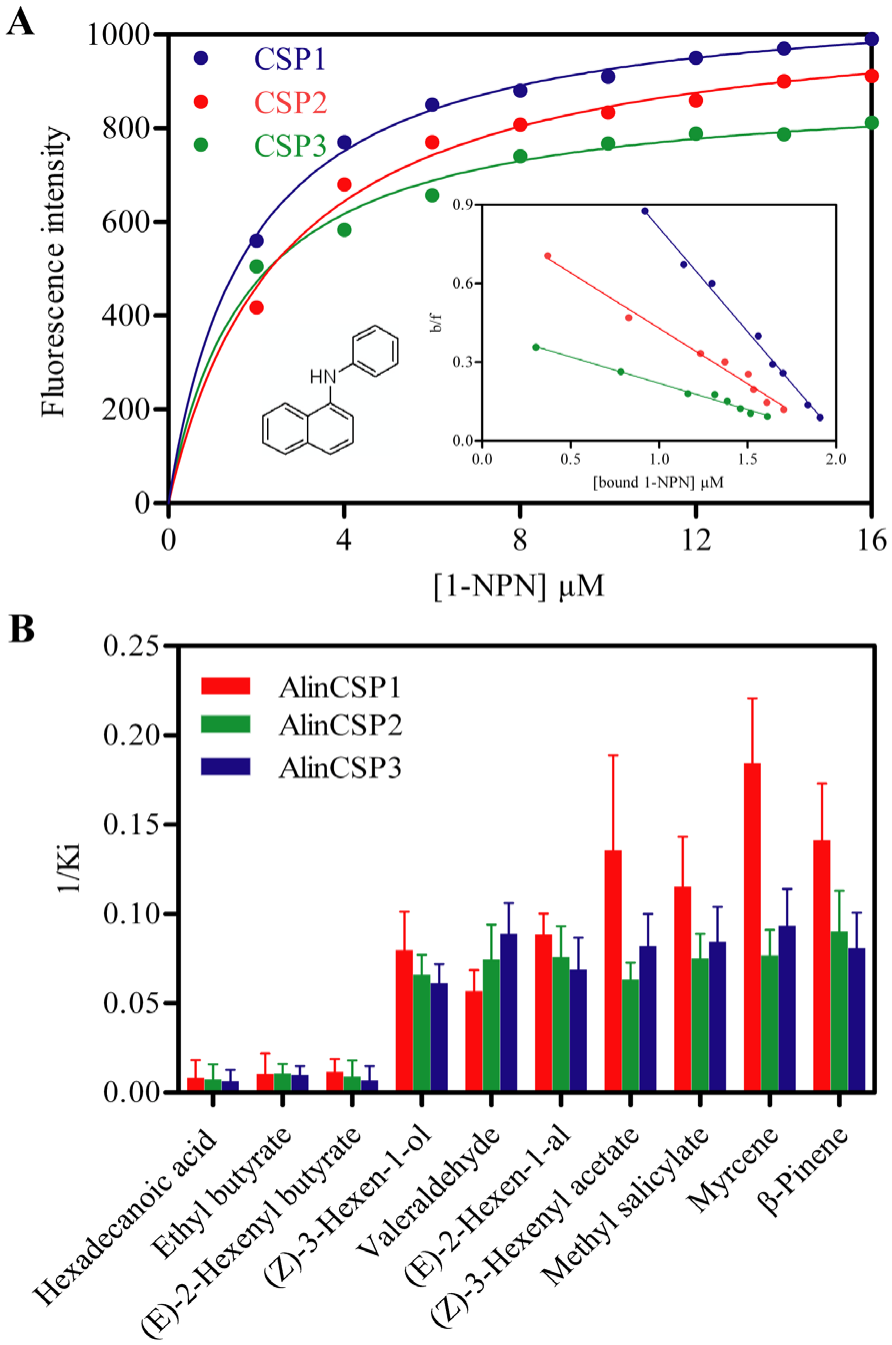
Fluorescence binding assay for the binding affinity of the plant bug CSPs. (A) Binding of 1-NPN to AlinCSP1, AlinCSP2 and AlinCSP3**.** The scatchard plot (the insert) indicate the binding constants of AlinCSP/1-NPN complex were 1.83 µM, 1.92 µM and 4.45 µM for AlinCSP1, AlinCSP2 and AlinCSP3, respectively. (B) The binding constants (K_i_ as presented as 1/K_i_) of the plant bug CSPs to selected ligands. The K_i_ values of AlinCSP1, AlinCSP2 and AlinCSP3 are 117.7 µM, 137.0 µM, 156.3 µM to hexadecanoic acid, respectively; 95.2 µM, 92.5 µM, 101.3 µM to ethyl butyrate, respectively, and 86.5 µM, 112.9 µM, 148.1 µM to (*E*)-2-hexenyl butyrate, respectively. The K_i_ values of other ligands are showed in Table 1. The binding curves for each of the CSPs and the ligand structures are shown in Figure S4.

AlinCSP2 exhibited an emission peak at 330 nm when excited at 295 nm, indicating Try 97 is located inside position of AlinCSP2 protein and in a relatively hydrophobic environment. With addition of 1-NPN, the intrinsic fluorescence was quenched by 1-NPN in a dose-dependent manner (Figure S3), indicating 1-NPN was bound inside of AlinCSP2. The dissociation constants of the AlinCSP/1-NPN complexes were 1.83 µM, 1.92 µM and 4.45 µM for AlinCSP1, AlinCSP2 and AlinCSP3 respectively (Figure 7A), which were used to calculate the dissociation constants (K_D_) of ligands.

The binding affinities of the three plant bug CSPs to 55 ligands are listed in Table 1 and shown in Figure 7B. The competitive binding curves of selected ligands to AlinCSP1-3 were shown in Figure S4. All three CSPs bound strongly with K_D_ values of 12.54 µM, 15.15 µM, 16.30 µM, respectively, to one of five alcohols, (*Z*)-3-hexen-1-ol, a volatile released by the *A. lineolatus* host plant alfalfa, *Medicago sativa* L. [3], [4]. The three CSPs also showed strong binding to two of five aldehydes, valeraldehyde and (*E*)-2-hexen-1-al with K_D_ values from 11.25 µM to 17.62 µM. The CSPs showed medium or week binding to four ketones and 11 esters, expect (*Z*)-3-hexenyl acetate which showed significant binding affinities to all three CSPs with K_D_ values of 7.37 µM, 15.82 µM and 12.16 µM for AlinCSP1, AlinCSP2 and AlinCSP3, respectively.

**Table 1:** Binding affinities of 55 chemical compounds to AlinCSP1-3 proteins.

	K_D_ (µM)				K_D_ (µM)		
Ligands	AlinCSP1	AlinCSP2	AlinCSP3	Ligands	AlinCSP1	AlinCSP2	AlinCSP3
**Aliphatic alcohols**				Ethyl heptanoate	43.16±2.21	38.29±1.57	35.26±2.51
2-Hexanol	45.36±2.43	u.d.^1^	37.09±1.01	**Aromatic compounds**			
2-Ethyl-1-hexanol	u.d.	u.d.	u.d.	Benzaldehyde	27.53±1.96	28.02±2.11	24.02±1.76
2-Octanol	u.d.	39.47±2.11	u.d.	Methyl salicylate	8.67±1.92	13.32±1.71	11.83±1.89
(Z)-3-Nonen-1-ol	21.48±1.17	26.43±1.87	25.54±1.67	3,4-Dimethyl-benzaldehe	u.d.	u.d.	u.d.
(Z)-3-Hexen-1-ol	12.54±2.32	15.15±1.79	16.30±1.98	Methyl phenylacetate	32.12±1.85	30.56±1.92	22.18±0.87
2-Undecanol	u.d.	u.d.	u.d.	2,3-Dimethylbenzoic acid	u.d.	u.d.	u.d.
Tetradecanol	u.d.	35.78±3.01	u.d.	Ethyl phenylacetate	27.31±2.02	25.26±1.98	24.40±1.32
**Aliphatic aldehydes**				**Heterocyclic compound**			
Valeraldehyde	17.62±2.54	13.42±2.41	11.25±1.52	Indole	27.12±1.91	22.42±2.01	24.65±2.16
(E)-2-Hexen-1-al	11.32±1.07	13.16±2.05	14.52±2.59	**Aliphatic terpenoids**			
Nonanal	43.65±1.11	u.d.	u.d.	Isoborneol	24.16±1.42	21.11±2.04	28.59±1.68
Decanal	u.d.	45.87±1.71	42.30±2.76	(-)-β-Citronellol	u.d.	u.d.	43.47±0.99
Dodecanal	u.d.	u.d.	u.d.	Citral	u.d.	u.d.	u.d.
**Saturated fatty acid**				Myrcene	5.42±0.93	13.04±1.72	10.71±1.64
hexadecanoic acid	u.d.	u.d.	u.d.	α-Terpinene	u.d.	u.d.	45.50±2.06
**Aliphatic ketones**				(+)-α-Pinene	31.71±0.98	30.62±0.71	27.19±1.02
2-Hexanone	19.51±1.73	20.41±1.82	22.46±1.88	β-Pinene	7.07±1.41	11.07±1.91	12.37±2.09
2-Heptanone	42.92±2.89	u.d.	41.23±2.56	Linalool	u.d.	46.21±2.35	u.d.
2-Octanone	u.d.	u.d.	39.49±1.91	(Z)−ocimene	28.64±1.71	25.13±0.93	22.15±1.01
2-Nonanone	u.d.	42.43±2.21	u.d.	Limonene	u.d.	u.d.	u.d.
**Aliphatic esters**				β-Caryophyllene	9.22±1.08	u.d.	12.35±2.42
Ethyl butyrate	u.d.	u.d.	u.d.	α-Humulene	9.96±0.89	u.d.	8.94±2.97
(Z)-3-Hexenyl acetate	7.37±2.49	15.82±1.68	12.16±1.82	Nerolidol	28.15±1.26	24.38±1.36	22.43±1.33
Butyl acetate	26.56±1.87	41.24±2.89	34.51±2.79	**Aliphatic alkanes**			
Butyl butyrate	u.d.	u.d.	u.d.	Pentane	42.21±1.95	39.46±2.54	35.62±2.61
Ethyl heptanoate	25.32±2.54	45.32±2.69	38.28±1.67	Octane	42.15±1.42	44.36±1.36	45.40±1.54
Hexyl butyrate	46.84±1.85	u.d.	u.d.	Nonane	u.d.	u.d.	u.d.
(E)-2-Hexenyl butyrate	u.d.	u.d.	u.d.	decane	44.55±0.75	46.42±0.82	47.23±1.18
Nonyl acetate	23.55±1.60	29.16±2.19	46.51±3.07	Undecane	38.45±1.54	39.55±2.36	u.d.
Hexyl hexanoate	44.66±2.38	u.d.	43.79±1.98	Dodecane	u.d.	u.d.	u.d.
Butyl acrylate	38.54±1.69	28.14±2.37	40.17±2.88	Tetradecane	u.d.	u.d.	46.18±2.76

1 u.d. indicates that the dissociation constants were not to be calculated if the IC_50_>50 µM. The company, CAS number and purity of all the tested chemicals are listed in Table S1.

The three CSPs also showed high binding affinities to methyl salicylate among the six aromatic compounds with K_D_ values of 8.67 µM, 13.32 µM and 11.83 µM for AlinCSP1, AlinCSP2 and AlinCSP3, respectively. Furthermore among 13 terpenoids, myrcene and β-pinene effectively displaced 1-NPN with K_D_ values from 5.42 µM to 13.04 µM for the CSPs. While other plant volatiles such as β-caryophyllene and α-humulene failed to bind with AlinCSP2, but bound to AlinCSP1 and AlinCSP3 with high binding affinity with the K_D_ values from 8.94 µM to 12.35 µM. All the seven alkanes failed to bind or showed very weak binding abilities with the three CSPs with the IC_50_>40 µM.

### Antenna Activity of Selected Semiochemicals

**Figure 8:**
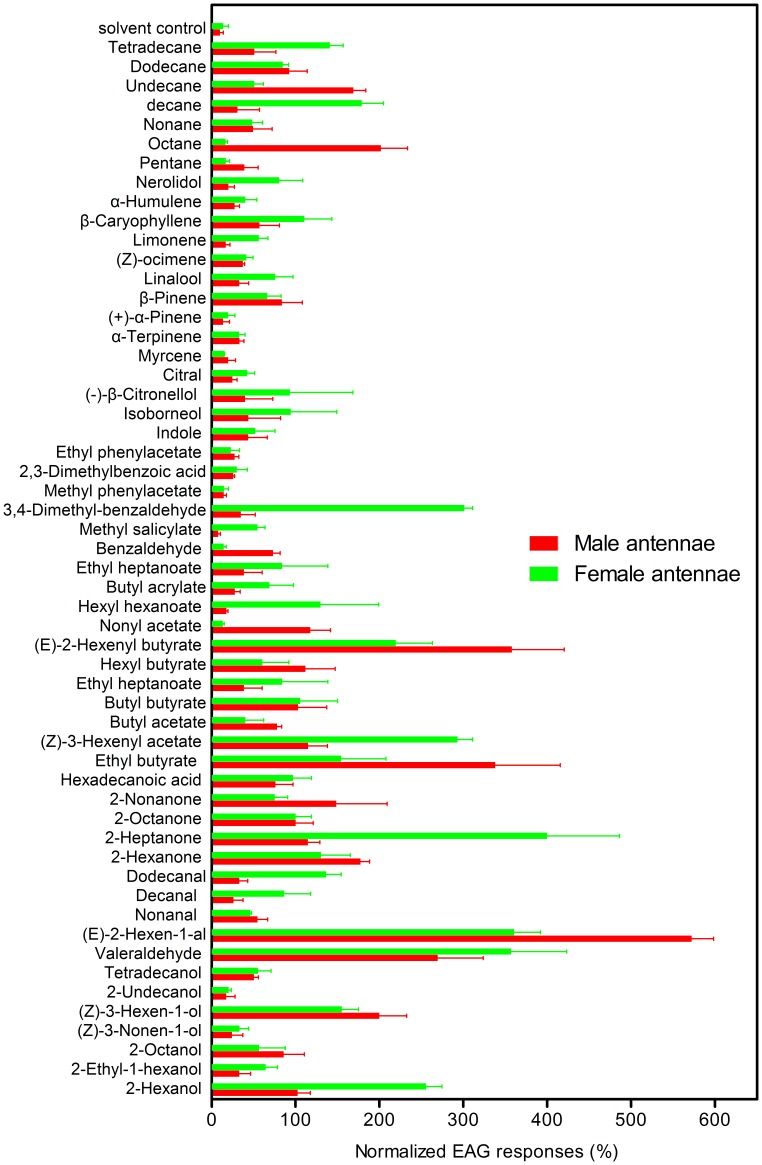
EAG responses in the antennae of male and female adults *A. lineolatus* to 55 chemicals. Each sample was tested three times against at least six insects. The averaged response for each insect was subtracted from the average response to a solvent blank and normalized with respect to the response 2-octanone.

Fifty five cotton plant volatiles and plant bug potential sex pheromones were selected to conduct the electroantennogram (EAG) recordings. The compounds which elicited more than 50% of 2-octanone EAG response regarded as good ligands. There were five compounds, (*Z*)-3-hexen-1-ol, valeraldehyde, (*E*)-2-hexen-1-al, ethyl butyrate and (*E*)-2-hexenyl butyrate elicited significant antennal responses from both male and female *A. lineolatus* (Figure 8) (p>0.05 for (*Z*)-3-hexen-1-ol, valeraldehyde and ethyl butyrate, p<0.05 for (*E*)-2-hexen-1-al and (*E*)-2-hexenyl butyrate). It is also noteworthy that 2-hexanone, octane, undecane elicited significantly higher EAG responses from male antennae than from female antennae (p>0.05 for 2-hexanone and p<0.001 for octane and undecane). 2-hexanol, 2-heptanone, (*Z*)-3-hexenyl acetate, 3,4-dimethyl-benzaldehyde and decane elicited significantly higher EAG responses from female antennae than from male antennae (p<0.001) (Figure 8).

### CSP Protein Structural Analysis

**Figure 9:**
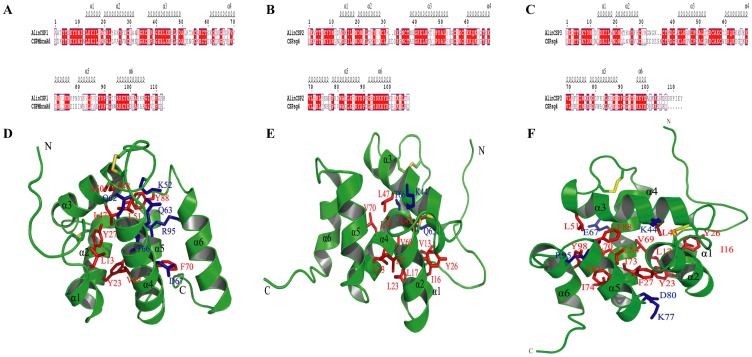
Predicted Structures of the CSPs of the alfalfa plant bug *A. lineolatus*. (A–C) Alignments between the plant bug CSP AlinCSP1 (A), AlinCSP2 (B) and AlinCSP3 (C) and the structural templates used in the homologous modeling. The secondary structure elements for the plant bug CSPs are shown on the top of the sequences. α-helices are displayed as squiggles. Strictly identical residues are highlighted in white letters with a red background. Residues with similar physico-chemical properties are shown in red letters. Alignment positions are framed in blue if the corresponding residues are identical or similar. (D-F) Cartoon representation of the plant bug CSPs AlinCSP1 (D), AlinCSP2 (E) and AlinCSP3 (F). Helices and two termini are labeled. Residues surrounding the binding pocket are shown as stick, where hydrophobic and hydrophilic residues are colored red and blue, respectively. Disulphide bridges are colored yellow.

To predict the 3D structures of the plant bug CSPs AlinCSP1-3, the 3D-Jury method [36] was employed to search for the structural templates. CSPMbraA6 of the moth *Mamestra brassicae* (PDB code: 1K19) [37] was chosen as template of AlinCSP1. CSPsg4 of the desert locusts *Schistocerca gregaria* (PDB code: 2gvs) [38] was used as template of AlinCSP2 and AlinCSP3. The sequence identity between AlinCSP1 and CSPMbraA6 is 48.2%, the sequence identity between AlinCSP2, AlinCSP3 and CSPsg4 is 56.9% and 48.6%, respectively (Figure 9A–C). Using the sequence alignments, the predicted 3D model of AlinCSP1-3 was generated with Modeler (Figure 9D–F) [39]. The Verify Score of the final AlinCSP1-3 model by Profiles-3D was 34.39, 48.28 and 36.12, respectively, which is much higher than expected score (22.75, 21.7 and 21.1 for AlinCSP1-3, respectively), implying that the overall quality of the predicted AlinCSP1-3 structure was generally reliable.

The predicted 3D structure of AlinCSP1-3 consisted of six a-helices and connected by α-α loops and two pairs of non-interlocked disulphide bridges in the pattern of Cys29-Cys36 and Cys55-Cys58, which formed two small loops (Figure 9D–F). The 3D model of AlinCSP1-3 revealed a large binding pocket, and most of the residues were hydrophobic, including leucine, proline, alanine, valine, phenylalanine, isoleucine and tyrosine. However, several hydrophilic residues, including lysine, glutamine, threonine, aspartic acid, arginine, glutamic acid, were also present in the binding pocket (Figure 9D–F), which might contribute to the formation of hydrogen bonds with the functional groups of some ligands.

## Discussion

The chemosensory proteins AlinCSP1-3 of the alfalfa plant bug *A. lineolatus* showed very high sequence identities (about 55%) with no-Hemipteran CSPs (Figure 2), supporting the view that insect CSPs are highly conserved even across very distant species [17], [18], [32] and implying important roles they might play in insect physiology. The constructed 3D structures of AlinCSP1-3 are very similar with other previously known insect CSP structures. Like the CSPMbraA6 of *Mamestra brassicae* and the CSPsg4 of the *Schistocerca gregaria*, the plant bug CSPs AlinCSP1-3 featured a hydrophobic binding pocket, the ligand binding differences may be due to some specific amino acid located in the hydrophobic cavities [38]. For example, in the CSPsg4, the Ile76 and Trp83 are involved in oleamide binding [38], in the CSPMbraA6, Tyr26 plays key role in the binding of 12-bromo-dodecanol (BrC12OH) [40]. So those amino acid residues located in the binding pocket of AlinCSP1-3, such as lysine, glutamine, threonine, aspartic acid, arginine, glutamic acid, may also be involved in the recognition and binding of the hydrophobic ligands. Similar key amino acids in the binding sites are also observed in the hydrophobic cavities of OBPs, for instance Ser56 in BmorPBP [34], Asn53 in ApolPBP1 [41], Glu98 in BmorGOBP2 [42], Asn74 in LmigOBP1 [43]. Further examination such as site-directed mutagenesis would be useful to evaluate these residues against with the semiochamicals that were identified in this study, showed a high binding affinity to the CSPs and elicited higher antenna activity in the plant bug.

Insect mainly use antennae to detect chemical stimuli from the environment [44]. Different olfactory sensilla in the antennae play a crucial role in host plant recognition and mate selection [45]. The CSP genes highly expressed in antennae has been proposed to regulate rapid switch between attraction and repulsion behaviors in the migratory locust [46]. The CSP genes that were mainly expressed in the female antennae and their transcript levels were increased remarkably after blood meal in the tsetse fly *Glossina morsitans morsitans*, were proposed to relate to the female host-seeking behavior [19]. Our qRT-PCR results revealed the three plant bug CSP genes *AlinCSP1-3* were highly expressed in *A. lineolatus* antennae. Interestingly, these three *CSP* genes were also expressed in the 5^th^ instar nymphs; these results suggest a function of AlinCSP1-3 in chemoreception and in maintaining particular life activities of the plant bug. The expression of AlinCSP1-3 proteins at high level in sensillar lymph of the pheromone-sensitive sensilla trichodea and general odorant-sensitive sensilla basiconica supports that AlinCSP1-3 protein may play important roles in *A. lineolatus* olfactory reception, strongly suggesting that antenna specific CSPs may play a role in insect chemoperception. However, these CSPs AlinCSP1-3 are not extremely selective and have binding affinity for the majority of compounds tested (Table1) as well as a wide presence in almost all types of sensilla. It is possible that these CSPs interact with other olfactory proteins after ligand binding in a sensillum and then such protein-protein interactions are recognized by olfactory receptors; in this case the CSPs expressed in pheromone sensitive sensilla might be indirectly involved in pheromone perception. Conversely and almost as likely, these plant bug CSPs function as a carrier to capture and transport semiochemicals to the membrane-bounded olfactory receptors. The binding of a ligand to the CSPs may trigger structural changes of the CSPs which are then recognized by the olfactory receptors either with or without the ligand resulting in action potentials. These combinatory activities of CSPs and olfactory receptors in a particular sensillum and the integration with other incoming signals from other sensilla at higher levels of the neuron system give insect extraordinary ability to discriminate between and response to particular host odors and sex pheromone components.

We could not exclude other roles these CSPs might play in the plant bug. The CSP from *Locusta migratoria* (LmigCSP-II) was detected in the sensilla chaetica of the wings and was suggested to be involved in contract chemoreception process [31]. In the moth *Cactoblastis cactorum*, the OS-D homolog CLP1 was mainly expressed in the female moth labial palps and was suggested that this protein is involved in carbon dioxide detection [25]. In the *Periplaneta Americana* (American cockroach), a CSP like gene named *P10* was expressed 30 times higher in the regenerating legs than in normal legs, indicating *P10* gene may be involved in the regeneration of insect legs [28], [29]. CSPs expressed in the proboscis, antennae and pheromonal glands of cabbage armyworm, *Mamestra brassicae* bound with sex pheromone analogues, suggest these CSPs may be devoted to pheromone detection [22], [27].

The mirids including *A. lineolatus* responded preferentially to flowering plants especially cotton, alfalfa and mung bean [3], [4], [6]-[8]. (*Z*)-3-Hexen-1-ol and (*E*)-2-hexen-1-al are volatiles released by alfalfa, *Medicago sativa* L. [7], which reported as main host plants of *A. lineolatus* [3]. Valeraldehyde was a volatile of cotton [47]. These three ligands showed very high binding affinities to AlinCSP1-3, and also elicited high EAG response in male and female *A. lineolatus* antennae. (*Z*)-3-Hexenyl acetate, is a volatile released by cotton when the plants damaged by herbivores [48], [49], field experiments showed that (*Z*)-3-hexenyl acetate efficiently attracted cotton mirids [50]. The binding assays showed AlinCSP1-3 bound (*Z*)-3-hexenyl acetate with high affinities, but only elicited a high EAG response in the female *A. lineolatus* antennae. These data further support a potential role of the CSPs AlinCSP1-3 in host recognition and also provide further evidence that CSPs may selectively capture and transport particular ligands to olfactory receptors.

Methyl salicylate was reported as a common component of insect-induced plant volatiles and supposed to help predators to find their prey [56], [57]. Myrcene, β-pinene were detected from alfalfa when the plants were damaged by western tarnished plant bug *Lygus hesperus Knight* [7], the binding experiments showed these three compounds had high binding abilities with AlinCSP1-3, but failed to elicit high EAG response in male and female *A. lineolatus* antennae. β-caryophyllene and α-humulene were reported as volatile compounds emitted from the cotton when the plants attacked by herbivorous insects [7], [47], [48], the binding assays showed these two chemicals had high binding affinities with AlinCSP1 and AlinCSP3, but no notable EAG response was observed with these two compounds with the plant bug antennae. These displacements between biological activities and biochemistry observations are our next challenges in the insect olfaction research.

The results of fluorescence displacement binding assay are not always correlated well with the biological activity of ligands tested. 2-Hexanone, octane, undecane elicited significantly higher EAG responses from the male antennae, and 2-hexanol, 2-heptanone, 3,4-dimethyl-benzaldehyde and decane elicited significantly higher EAG responses from the female antennae, however, none of these compounds showed a good affinity to any one of the three CSPs AlinCSP1-3. Furthermore, hexadecanoic acid was a main volatile released by mungbean, a trap crop of pest mirids [8], [51], but this compounds ineffectively bound with AlinCSP1-3 with IC_50_>50 µM and did not elicit notably EAG responses. Similarly, ethyl butyrate and (*E*)-2-hexenyl butyrate were reported as two major sex pheromone components of most plant bugs [52]–[55], they elicited high EAG responses, however, failed to bind with any of the three CSPs. It is very likely that there are more CSP and OBP genes in the plant bug genome and the cDNA library screening approach failed to detect them. The CSPs encoded by these genes would be capable of capturing and transporting them to olfactory receptors. This would also indicate that AinCSP1-3 may not be the sex pheromone binding proteins of the plant bug and unlikely participate in the sex pheromone reception progress.

In conclusion, the antennal specific expression of the CSP genes and proteins, the high affinity binding to biological active semochemicals support a possible functional role of the chemosensory proteins AlinCSP1-3 in the perception of general odorants but not sex pheromones of *A. lineolatus*. And thus the three antennae-biased CSPs may mediate host recognition in the *A. lineolatus* and represent new interesting targets for the control of their population in agriculture.

## Materials and Methods

### Insect Rearing

The *A. lineolatus* nymphs and adults were collected from cotton fields at the Langfang Experimental Station of Chinese Academy of Agricultural Sciences, Hebei Province, China. A laboratory colony was established and maintained at 29±1°C, 60±5% relative humidity (RH), and 14∶10 light:dark (L:D) and reared on green beans and a 10% sucrose solution.

### Screening of CSP Genes in the Antennal cDNA Library

Total antennal RNA was isolated by trizol reagent (Invitrogen, Carlsbad, CA, USA). The antennal cDNA library was constructed using the Creator SMART cDNA Library Construction Kit (Clontech, Mountain, CA, USA). Detailed protocol of library construction was followed Gu et al. [58]. Single clones were picked and sequenced with standard M13 primers using the ABI3730XL sequencer (Applied Biosystems, Carlsbad, CA, USA). Genes encoding candidate CSP genes were identified by BlastX and the “CSP MotifSearch program” of C_1_-X_6-8_-C_2_-X_16-21_-C_3_-X_2_-C_4_ [32].

### CSP Protein Sequences and Structural Analysis

CSPs protein sequences identified in *A. lineolatus* and reported in other insect species were aligned using ClustalX 1.83 [59]. The putative N-terminal signal peptides were predicted by the SignalP V3.0 program [60] (http://www.cbs.dtu.dk/services/SignalP/).

A 3D-Jury method [36] was used to search structural templates of AlinCSP1-3. Several identified CSP protein structures were used as templates to construct 3D structures of AlinCSP1-3 using the Modeler module [39] in Discovery Studio 2.0 (Accelrys Software Inc. San Diego, CA), the one with the highest score of Profiles-3D [61] was retained. The CHARMm [62] force field was employed to refine the initial homology model. The Profiles-3D method [61] and Ramachandran plot [63] were used to evaluate the rationality of the established 3D model.

### Quantitative RT-PCR (qRT-PCR)

Male antennae, female antennae, head (without antennae), thorax, abdomen, legs and wings of adult individuals were excised and immediately frozen in liquid nitrogen, and then stored at -80°C until use. The developmental stages of *A. lineolatus* were classified according to the criteria of Lu and Wu [4]. Total RNA of each sample was isolated by trizol regent. Before transcription, the RNA was treated with DNase I (Invitrogen, Carlsbad, CA) to remove residual genomic DNA. First strand cDNA was synthesized using the SuperScript™ III Reverse Transcriptase system (Invitrogen, Carlsbad, CA).

The ORF sequence close to the 5′end of the AlinCSP1-3 gene was used for designing PCR primers with the following critera: primers 18–25 bp in length, primer annealing temperature of 55–61°C and amplicon sizes of 80–150 bp. Two reference genes, β-actin (GenBank No. GQ477013) and elongation factor (GenBank No. JQ082478) were used in each qRT-PCR experiment. The qRT-PCR primers were designed using Beacon Designer 7.90 (PREMIER Biosoft International) and are:

AlinCSP1-F(62–84 bp): 5′- ACACGTCCAAATACGACAATATC -3′.

AlinCSP1-R(133–150 bp): 5′- CTTGTTGGCGAGGCAGTC -3′.

AlinCSP2-F(12–29 bp): 5′- CGTGTTGGTTCTATTGTG-3′.

AlinCSP2-R(88–107 bp): 5′- TCATTGGAGAGGATCTTATC-3′.

AlinCSP3-F(68–87 bp): 5′- ACACGGACAAATATGACAAT -3′.

AlinCSP3-R(131–150 bp): 5′- CATAACGCAGTGGAAATAGT -3′.

β-actin-F(1004–1021 bp): 5′- AACAAGAATACGACGAAT -3′.

β-actin-R(1136–1154 bp): 5′- GAATGGGAGAAATCAAATG -3′.

Elongation factor-F(963–980 bp): 5′- CTACACCATCGTACAAGA -3′.

Elongation factor-R(1019–1038 bp): 5′- GTCAAGATATTGCGTAAGAT -3′.

qRT-PCR experiments were performed using 96 well plates (Applied Biosystems, Carlsbad, CA), ABI Prism 7500 Fast Detection System (Applied Biosystems, Carlsbad, CA) and Brilliant II SYBR Green qPCR master mix (Stratagene, La Jolla, CA). qRT-PCR was conducted in 25 µl reactions containing 2×Brilliant II SYBR Green qPCR master mix 12.5 µl, primer (15 µM) 1 µl, passive reference dye 0.375 µl (20 µM), sample cDNA 1 µl, sterilized H_2_O 9.215 µl. Cycling conditions were: 50°C for 20 s, 95°C for 10 min, 40 cycles of 95°C for 15 s and 60°C for 1 min. Afterwards, the PCR products were heated to 95°C for 15 s, cooled to 60°C for 1 min and heated to 95°C for 30 s and cooled to 60°C for 15 s to measure the dissociation curves. No-template and no-reverse transcriptase controls were included in each experiment. To check reproducibility, each test sample was done in triplicate technical replicates and two biological replicates.

### qRT-PCR Data Analysis

Raw Ct values were converted to quantities representing relative expression levels using a modified comparative Ct method [64], with correction for different amplification efficiencies [65]. Briefly, after qRT-PCR, Ct values were exported into the LinRegPCR program to correct the amplification efficiencies for each reaction. The relative expression levels (Pfaffl ratio) of AlinCSP genes to the reference gene was then calculated for each sample as: E_csp_^ΔCt,CSP^_/_E_ref_^ΔCt,ref^.

Where E_csp_ and E_ref_ are corrected amplification efficiencies for the AlinCSP and reference gene, respectively, and in different tissues,

ΔCt,CSP is calculated as: Ct,_CSP of abdomen_ - Ct,_CSP of X._

And ΔCt,ref is calculated as: Ct,_ref of abdomen_ - Ct,_ref of X._

And In different development stages,

ΔCt,CSP is calculated as: Ct,_CSP of 1 st instar_ - Ct,_CSP of X._

And ΔCt,ref is calculated as: Ct,_ref of 1 st instar_ - Ct,_ref of X._

Where ref represents β-actin or elongation factor gene, X represents different tissues or different development stages.

In the analysis of the relative fold change in different tissues (or different stages), the abdomen (or 1^st^ instar) sample was taken as the calibrator. Thus, the relative fold change in different tissues (or different stages) was assessed by comparing the expression level of AlinCSPs in other tissues (or development stages) with that in the abdomen (or 1^st^ instar).

### Expression and Purification of Recombinant AlinCSP1-3 Proteins

Gene specific primers are designed to clone the coding region of AlinCSP1-3 and as followed:

AlinCSP1-F: 5′- GTCATATGGCTGCCACGTACACGTCC -3′.

AlinCSP1-R: 5′-TGAAGCTTTCAGTGGCTTCCGGGCAA-3′.

AlinCSP2-F: 5′- GTCATATGGCTGAAGTTTACACTTCG-3′.

AlinCSP2-R: 5′-TGAAGCTTTTAAGCAGTGAGAGCTTT-3′.

AlinCSP3-F: 5′-GTCATATGGCGGAGCAGTACACGGAC-3′.

AlinCSP3-R: 5′-TGAAGCTTTTAGTATTCAATAGGTTT-3′.

(Underlined showed *Nde* I and *Hind* III enzyme sites in the forward and reverse primer, respectively.)

The PCR products were first cloned into pGEM-T easy vector (Promega, Madison, WI) and then excised and cloned into the bacterial expression vector pET30a(+) (Novagen, Madison, WI) between the *Nde* I and *Hind* III restriction sites, and verified by sequencing. Plasmid containing the correct insert was extracted and transformed into *E.coli* BL21(DE3) competent cells. A verified single colony was grown overnight in 50 ml LB broth (including 100 µg/ml Kanamycin). Five liters of LB medium was inoculated with the 50-ml overnight culture at 37°C for 2–3 hours until the absorbance at OD_600_ reached 0.6. The proteins were then induced with isopropyl-β-D-thiogalactopryranoside (IPTG) with a final concentration of 1 mM at 28°C for 8 hours. The bacterial cells were harvested by centrifugation (8000 g, 10 min), resuspended in the lysis buffer (80 mM Tris-HCl, 200 mM NaCl, 1 mM EDTA, 4% glycerol, pH 7.2, 0.5 mM PMSF), lysed by sonication (10 sec, 5 passes) and centrifuged again (12000 g, 10 min). The soluble fraction and the whole pellet were analyzed by sodium dodecyl sulfate polyacrylamide gel electrophoresis (SDS-PAGE) and found the CSP proteins mainly present in the inclusion bodies. Insoluble protein were washed with 0.2% triton X-100 in 50 mM Tris buffer (PH 6.8) and then dissolved in 6 M guanidinium hydrochloride, the protein refolding protocols performed using the redox methods as described by Prestwich [66].

Soluble and refolded CSP protein was purified by anion-exchange chromatography with two rounds of HiTrap Q HP anion exchange columns (GE Healthcare Biosciences, Uppsala, Sweden), and one round of Mini Q 4.6/50 anion exchange columns (GE Healthcare Biosciences, Uppsala, Sweden), and two round of gel filtration on a Superdex 75 10/300 GL column for the final purification (GE Healthcare Biosciences, Uppsala, Sweden). Highly purified protein fractions were desalted by HiTrap Desalting Columns (GE Healthcare Biosciences, Uppsala, Sweden) and then concentrated using Amicon 10 KDa cutoff concentrators (Millipore). The size and purity of AlinCSP3 were checked by SDS-PAGE and mass spectroscopy analysis. The concentration of the purified AlinCSP1-3 protein was measured by the Bradford method using BSA as standard protein [67].

### Preparation of Antisera

AlinCSP1-3 antisera were obtained by injecting adult male rabbits subcutaneously and intramuscularly. The protein was emulsified with an equal volume of Freund’s complete adjuvant for the first injection and incomplete adjuvant for further injection. Blood was collected 7 days after the last injection and centrifuged at 6000 rpm for 20 min. The supernatant serum was further purified by precipitation in 40% ammonium sulphate and then purified by Protein A affinity chromatography method.

### Scanning Electron Microscopy (SEM)

Antennae were cut from newly emergence male and female *A. lineolatus.* The antennae sample were first fixed in 70% ethanol for 3 hours and then cleaned in an ultrasonic bath (250W) for 15 seconds. After gradient elution in an ethanol series (80%, 90%, 95% and 100%), the antennae sample were dried in 25°C oven thermostat for 10 hours. Samples were mounted on holders and viewed using a HITACHI S570 SEM (Hitachi Ltd., Tokyo, Japan) after coated with gold-palladium. During sputtering, the chamber pressure was 12 KV. Different sensilla types were classified according to the criteria of reported previously [35], [44].

### Immunocytochemical Localization

Antennae of male and female adult *A. lineolatus* were chemically fixed in a mixture of paraformaldehyde (4%) and glutaraldehyde (2%) in 0.1 M PBS (pH = 7.4) at 4°C overnight, then dehydrated in an ethanol series and embedded in LR White resin (Taab, Aldermaston, Berks, UK). Ultrathin sections (60–80 nm) were treated with primary antisera (anti-AlinCSP1-3) diluted at 1∶5000–1∶10000 at 4°C overnight. The secondary antibody was anti-rabbit IgG conjugated with 10 nm colloidal gold granules (Sigma, St. Louis, MO) at a dilution of 1∶20 and incubated with sections at room temperature for 60 min. Optional silver intensification [68] was used to enlarge the size of the gold granules to 30–40 nm. Sections were stained with 2% uranyl acetate to increase the contrast and observed in HITACHI H-7500 transmission electron microscopy (Hitachi Ltd., Tokyo, Japan). Labeling intensities were observed in three male and three female adult antennae.

### Fluorescence-based Ligand Binding Assays

Fluorescence binding assays were performed on an F-380 fluorescence spectrophotometer (Tianjin, China) in a 1 cm light path quartz cuvette. The slit width used for excitation and emission was both 10 nm. The fluorescent probe N-phenyl-1-naphthylamine (1-NPN) was dissolved in methanol with a 1 mM stock solution. 1-NPN was excited at 337 nm and emission spectra were recorded between 390 nm and 530 nm. The company, CAS number and purity of the 55 tested chemicals used in the binding assays are listed in Table S1.

The AlinCSP2 protein can produce intrinsic fluorescence for a single tryptophan at position 97. The tryptophan intrinsic fluorescence was measured with 5 µM AlinCSP2 protein in 50 mM Tris-HCl buffer, pH 7.4. The excitation wavelength was 295 nm and the emission spectrum was recorded between 310 and 450 nm. Quenching of intrinsic fluorescence was measured in the same conditions in the presence of 1-NPN at concentrations of 5, 10, 15 and 20 µM, respectively.

To measure the affinity of 1-NPN to AlinCSP1-3, a 2 µM solution of protein in 50 mM Tris-HCl, PH 7.4, was titrated with aliquots of 1 mM 1-NPN to a final concentration between 2 and 16 µM. The affinities of the 55 chemicals were measured by competitive binding assays, using both 1-NPN and AlinCSP1-3 at 2 µM by adding ligands from 2 to 16 µM. All values reported were obtained from three independent measurements.

### Binding Data Analysis

For determining binding constants, the intensity values corresponding to the maximum fluorescence emission were plotted against free ligand concentration. Bound ligand was evaluated from the values of fluorescence intensity assuming the protein was 100% active, with a stoichiometry of 1∶1 protein:ligand at saturation. The curves were linearized using Scatchard Plot. Dissociation constants of the competitors were calculated from the corresponding IC_50_ values, using the equation: K_D_  =  [IC_50_]/(1+[1-NPN]/K_1-NPN_), where [1-NPN] is the free concentration of 1-NPN and K_1-NPN_ is the dissociation constant of the AlinCSP1-3/1-NPN complex.

### Electrophysiological Recordings

Electrophysiological (EAG) recordings from each sex of adult individuals were made using Ag-AgCl glass pipette electrodes filled with 3 M KCl solution [69], [70]. The insects were anaesthetized by chilling, the antenna was amputated at the base and the tip of the antenna was cut off. The excised antenna was mounted between the electrodes by one teardrop of Spectra R360 electrically conductive gel (Syntech, Netherlands). The base of the antenna was connected to the reference electrode, while the terminal of tip-cut off antenna was connected to the recording electrode. An air stimulus controller CS-55 (Syntech, Netherlands) was used for air and chemical stimulants delivery with a constant flow of 10 ml/second flowed continuously over the antenna through the open end of the glass tube, which was positioned 1 cm from the antenna.

Twenty microliters of tested chemicals (100 µl/ml, 10% (v/v) dilution in paraffin oil except the hexadecanoic acid dissolved in hexane with 100 µg/ml) were applied to filter paper strips (1.5 cm×4 cm, Whatman No.1), and the solvent was allowed to evaporate for 30 sec before the paper strip was inserted into the glass Pasteur pipette cartridge (10 cm long, Fisher Scientific, Pittsburgh, Pennsylvania, USA). The chemicals were tested randomly, stimulus duration time was 0.5 second and an interval of at least 30 seconds was taken between stimulations for antennal recovery. Chemical odorants used for the EAG recordings are the same as used in the binding assays. Preliminary experiments showed that 2-octanone can elicit stable EAG signals and so used as a reference compound. The paraffin oil (Fluka, Buchs, Switzerland) was used to dissolve the chemicals and treated as control compound [71]. Each chemical was tested against six individual male and female antennae, and each antenna was tested three times. The signals were passed through a high impedance amplifier (CS-05 model, Syntech, the Netherlands), EAG responses were initially measured in millivolts (peak height of depolarization) and then converted to normalized responses by the Syntech EAG 2000 program (Syntech, the Netherlands). Response to the solvent control was subtracted from all normalized response and the normalized EAG responses were expressed as a percentage to the reference compound response [72]. All results are presented as mean (±SE) normalized EAG responses. EAG responses between male and female individuals were compared with the Student t-test using SAS software.

## Supporting Information

Figure S1 **The molecular weight and purity of AlinCSP1–3 protein were measured by matrix-assisted laser desorption/ionization (MALDI)-time-of-flight (TOF) mass spectrometers (Bruker Daltonics).**
(DOCX)

Figure S2 **The blue shift and increase in the fluorescence intensity when 1-NPN bound to AlinCSP1–3.** The fluorescence intensity of 5 µM 1-NPN in Tris-HCl buffer (pH = 7.4) was measured with the excitation wavelength at 337 nm. The blue shift was measured in the same conditions in the presence of 5 µM AlinCSP1 (A), AlinCSP2 (B), AlinCSP3 (C).
(DOCX)

Figure S3 **Intrinsic fluorescence and quenching effect of AlinCSP2.** The tryptophan intrinsic fluorescence was measured with 5 µM AlinCSP2 protein in 50 mM Tris-HCl buffer, pH 7.4. The excitation wavelength was 295 nm and the emission spectrum was recorded between 310and 450 nm. Quenching of intrinsic fluorescence was measured in the same conditions in the presence of 1-NPN at concentrations of 5, 10, 15 and 20 µM, respectively.
(DOCX)

Figure S4 **Competitive binding curves of selected ligands to AlinCSP1–3.** This figure showed the binding curves of 10 ligands to AlinCSP1, similar binding curves were obtained of AlinCSP2 and AlinCSP3. A mixture of the protein and 1-NPN in Tris buffer, pH = 7.4, both at the concentration of 2 µM, was titrated with aliquots of 1 mM methanol solutions of the ligands to final concentrations of 2–16 µM. Fluorescence values were tested as percent of the values in the absence of competitor. Data are means of three independent experiments. The binding abilities of AlinCSP1–3 protein with other ligands are listed in table 1.
(DOCX)

Table S1 **Company, CAS number and purity of all the tested chemicals.**
(DOCX)
