# Supplementary material for: Functional Characterizations of Chemosensory Proteins of the Alfalfa Plant Bug Adelphocoris lineolatus Indicate Their Involvement in Host Recognition
Source: PLoS One. 2012 Aug 10;7(8):e42871. doi: 10.1371/journal.pone.0042871 (PMC3416781; doi:10.1371/journal.pone.0042871)
Supplement: Figure S1 — The molecular weight and purity of AlinCSP1–3 protein were measured by matrix-assisted laser desorption/ionization (MALDI)-time-of-flight (TOF) mass spectrometers (Bruker Daltonics). (DOCX) [file pone.0042871.s001.docx]

**Figure S1. The molecular weight and purity of AlinCSP1-3 protein were measured by matrix-assisted laser desorption/ionization (MALDI)-time-of-flight (TOF) mass spectrometers (Bruker Daltonics).**

AlinCSP1

AlinCSP2

AlinCSP3
