# Supplementary material for: Functional Characterizations of Chemosensory Proteins of the Alfalfa Plant Bug Adelphocoris lineolatus Indicate Their Involvement in Host Recognition
Source: PLoS One. 2012 Aug 10;7(8):e42871. doi: 10.1371/journal.pone.0042871 (PMC3416781; doi:10.1371/journal.pone.0042871)
Supplement: Figure S2 — The blue shift and increase in the fluorescence intensity when 1-NPN bound to AlinCSP1–3. The fluorescence intensity of 5 µM 1-NPN in Tris-HCl buffer (pH = 7.4) was measured with the excitation wavelength at 337 nm. The blue shift was measured in the same conditions in the presence of 5 µM AlinCSP1 (A), AlinCSP2 (B), AlinCSP3 (C). (DOCX) [file pone.0042871.s002.docx]

**Figure S2. The blue shift and increase in the fluorescence intensity when 1-NPN bound to AlinCSPs.** The fluorescence intensity of 5 μM 1-NPN in Tris-HCl buffer (pH=7.4) was measured with the excitation wavelength at 337 nm. The blue shift was measured in the same conditions in the presence of 5 μM AlinCSP1 (A), AlinCSP2 (B), AlinCSP3 (C).


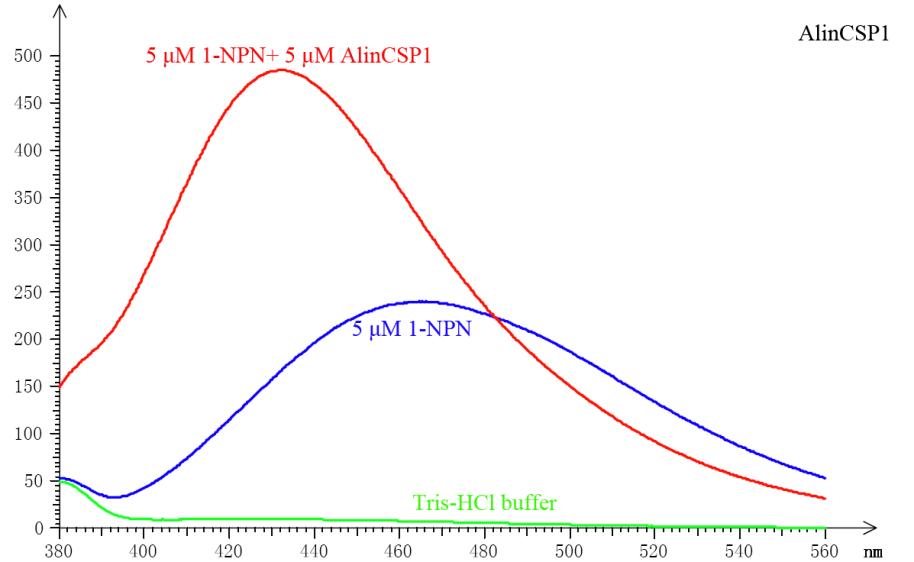


(A)


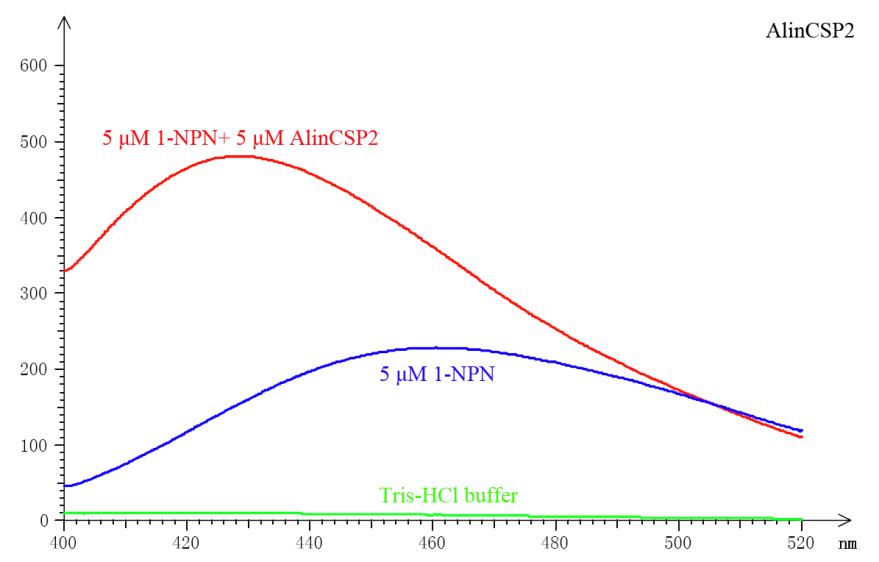


(B)


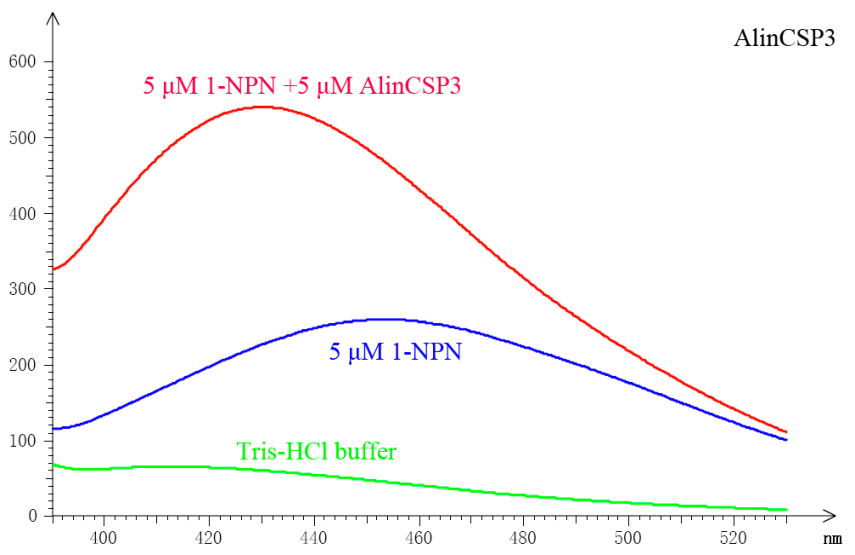


(C)
