# Supplementary material for: Functional Characterizations of Chemosensory Proteins of the Alfalfa Plant Bug Adelphocoris lineolatus Indicate Their Involvement in Host Recognition
Source: PLoS One. 2012 Aug 10;7(8):e42871. doi: 10.1371/journal.pone.0042871 (PMC3416781; doi:10.1371/journal.pone.0042871)

**Figure S3. Intrinsic fluorescence and quenching effect of AlinCSP2.** The tryptophan intrinsic fluorescence was measured with 5 μM AlinCSP2 protein in 50 mM Tris-HCl buffer, pH 7.4. The excitation wavelength was 295 nm and the emission spectrum was recorded between 310and 450 nm. Quenching of intrinsic fluorescence was measured in the same conditions in the presence of 1-NPN at concentrations of 5, 10, 15 and 20 μM, respectively.


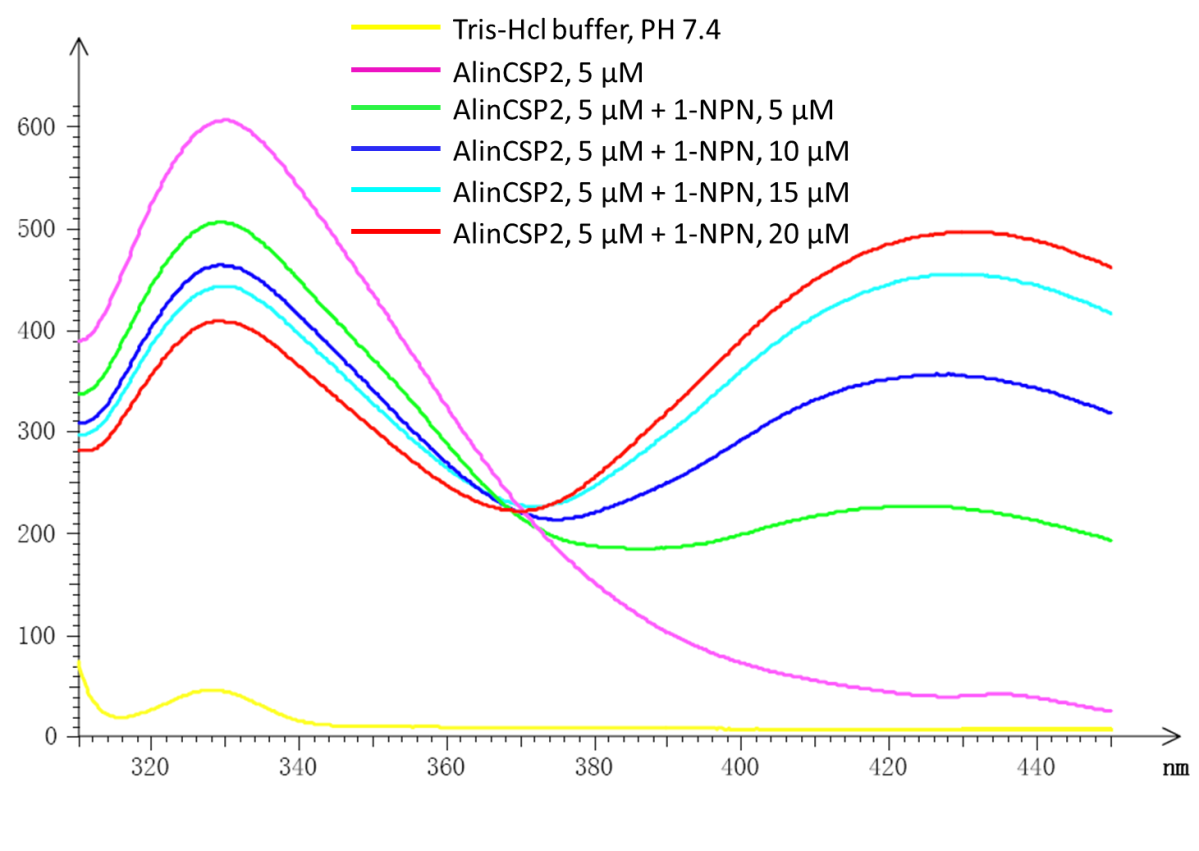

Supplement: Figure S3 — Intrinsic fluorescence and quenching effect of AlinCSP2. The tryptophan intrinsic fluorescence was measured with 5 µM AlinCSP2 protein in 50 mM Tris-HCl buffer, pH 7.4. The excitation wavelength was 295 nm and the emission spectrum was recorded between 310and 450 nm. Quenching of intrinsic fluorescence was measured in the same conditions in the presence of 1-NPN at concentrations of 5, 10, 15 and 20 µM, respectively. (DOCX) [file pone.0042871.s003.docx]
