# Supplementary material for: Functional Characterizations of Chemosensory Proteins of the Alfalfa Plant Bug Adelphocoris lineolatus Indicate Their Involvement in Host Recognition
Source: PLoS One. 2012 Aug 10;7(8):e42871. doi: 10.1371/journal.pone.0042871 (PMC3416781; doi:10.1371/journal.pone.0042871)
Supplement: Figure S4 — Competitive binding curves of selected ligands to AlinCSP1–3. This figure showed the binding curves of 10 ligands to AlinCSP1, similar binding curves were obtained of AlinCSP2 and AlinCSP3. A mixture of the protein and 1-NPN in Tris buffer, pH = 7.4, both at the concentration of 2 µM, was titrated with aliquots of 1 mM methanol solutions of the ligands to final concentrations of 2–16 µM. Fluorescence values were tested as percent of the values in the absence of competitor. Data are means of three independent experiments. The binding abilities of AlinCSP1–3 protein with other ligands are listed in table 1. (DOCX) [file pone.0042871.s004.docx]

**Figure S4. Competitive binding curves of selected ligands to AlinCSP1-3.** This figure showed the binding curves of 10 ligands to AlinCSP1, similar binding curves were obtained of AlinCSP2 and AlinCSP3. A mixture of the protein and 1-NPN in Tris buffer, pH=7.4, both at the concentration of 2 μM, was titrated with aliquots of 1mM methanol solutions of the ligands to final concentrations of 2-16 μM. Fluorescence values were tested as percent of the values in the absence of competitor. Data are means of three independent experiments. The binding abilities of AlinCSP1-3 protein with other ligands are listed in table 1.





β-Pinene

Myrcene

Methyl salicylate

(Z)-3-Hexenyl acetate

(E)-2-Hexenyl butyrate

(Z)-3-Hexen-1-ol

Valeraldehyde

Ethyl butyrate

(E)-2-Hexen-1-al

Hexadecanoic acid
