# Supplementary material for: Functional Characterizations of Chemosensory Proteins of the Alfalfa Plant Bug Adelphocoris lineolatus Indicate Their Involvement in Host Recognition
Source: PLoS One. 2012 Aug 10;7(8):e42871. doi: 10.1371/journal.pone.0042871 (PMC3416781; doi:10.1371/journal.pone.0042871)
Supplement: Table S1 — Company, CAS number and purity of all the tested chemicals. (DOCX) [file pone.0042871.s005.docx]

| **Chemicals** | **Company** | **CAS Number** | **Purity** | **Chemicals** | **Company** | **CAS Number** | **Purity** |
| --- | --- | --- | --- | --- | --- | --- | --- |
| **Aliphatic alcohols** |  |  |  | Ethyl heptanoate | Aldrich | 106-30-9 | ≥98% |
| 2-Hexanol | Aldrich | 626-93-7 | 99% | **Aromatic compounds** |  |  |  |
| 2-Ethyl-1-hexanol | Aldrich | 104-76-7 | ≥99.6% | Benzaldehyde | Fluka | 100-52-7 | ≥99% |
| 2-Octanol | Aldrich | 123-96-6 | 97% | Methyl salicylate | Aldrich | 119-36-8 | ≥98% |
| *cis*-3-Nonen-1-ol | Aldrich | 10340-23-5 | 95% | 3,4-Dimethyl-benzaldehyde | Aldrich | 5973-71-7 | 98% |
| *Cis*-3-Hexen-1-ol | Aldrich | 928-96-1 | ≥98% | Methyl phenylacetate | Aldrich | 101-41-7 | ≥98% |
| 2-Undecanol | Aldrich | 1653-30-1 | 97% | 2,3-Dimethylbenzoic acid | Aldrich | [603-79-2](http://www.sigmaaldrich.com/catalog/Lookup.do?N5=CAS+No.&N3=mode+matchpartialmax&N4=603-79-2&D7=0&D10=&N25=0&N1=S_ID&ST=RS&F=PR) | 98% |
| Tetradecanol | Fluka | 112-72-1 | ≥99.0% | Ethyl phenylacetate | Aldrich | 101-97-3 | ≥98% |
| **Aliphatic aldehydes** |  |  |  | **Heterocyclic compound** |  |  |  |
| Valeraldehyde | Aldrich | 110-62-3 | 97% | Indole | Aldrich | 120-72-9 | ≥99% |
| *trans*-2-Hexen-1-al | Aldrich | 6278-26-3 | ≥95% | **Aliphatic terpenoids** |  |  |  |
| Nonanal | Aldrich | 124-19-6 | ≥95% | Isoborneol | Aldrich | 124-76-5 | ≥95% |
| Decanal | Sigma | 112-31-2 | ≥98% | (-)-β-Citronellol | Fluka | 7540-51-4 | ≥98.5% |
| Dodecanal | Aldrich | 112-54-9 | 92% | Citral | Aldrich | 5392-40-5 | 95% |
| **Saturated fatty acid** |  |  |  | Myrcene | Aldrich | 123-35-3 | ≥90% |
| hexadecanoic acid | Sigma | 57-10-3 | ≧99% | α-Terpinene | Aldrich | 99-86-5 | ≥95% |
| **Aliphatic ketones** |  |  |  | (+)-α-Pinene | Aldrich | 7785-70-8 | ≥99% |
| 2-Hexanone | Fluka | 591-78-6 | 99.5% | β-Pinene | Aldrich | 18172-67-3 | ≥99% |
| 2-Heptanone | Aldrich | 110-43-0 | ≥98% | Linalool | Aldrich | 126-91-0 | ≥95% |
| 2-Octanone | Aldrich | 111-13-7 | 98% | (Z)−ocimene | Aldrich | 13877-91-3 | ≥90% |
| 2-Nonanone | Aldrich | 821-55-6 | ≥99% | Limonene | Sigma | 5989-27-5 | 97% |
| **Aliphatic esters** |  |  |  | β-Caryophyllene | Aldrich | 87-44-5 | ≥80% |
| Ethyl butyrate | Aldrich | 105-54-4 | 99% | α-Humulene | Aldrich | 6753-98-6 | ≥96% |
| *cis*-3-Hexenyl acetate | Aldrich | 3681-71-8 | ≥98% | Nerolidol | Aldrich | 7212-44-4 | 98% |
| Butyl acetate | Fluka | 123-86-4 | ≥99.7% | **Aliphatic alkanes** |  |  |  |
| Butyl butyrate | ACROS | 109-21-7 | 98% | Pentane | Fluka | 109-66-0 | ≥99% |
| Ethyl heptanoate | Fluka | 106-30-9 | ≥96% | Octane | Fluka | 111-65-9 | ≥99% |
| Hexyl butyrate | Aldrich | 2639-63-6 | ≥98% | Nonane | Sigma-Aldrich | 111-84-2 | ≥99% |
| *trans*-2-Hexenyl butyrate | Aldrich | 53398-83-7 | ≥96% | decane | Sigma-Aldrich | 124-18-5 | ≥99% |
| Nonyl acetate | Aldrich | 143-13-5 | 0.864g/ml | Undecane | Aldrich | 1120-21-4 | ≥99% |
| Hexyl hexanoate | Aldrich | 6378-65-0 | ≥97% | Dodecane | Sigma-Aldrich | 112-40-3 | ≥99% |
| Butyl acrylate | Fluka | 141-32-2 | ≥99.5%% | Tetradecane | Aldrich | 629-59-4 | ≥99% |

**Table S1. Company, CAS number and purity of all the tested chemicals.**
